# Supplementary material for: Cell Fate following Irradiation of MDA-MB-231 and MCF-7 Breast Cancer Cells Pre-Exposed to the Tetrahydroisoquinoline Sulfamate Microtubule Disruptor STX3451
Source: Molecules. 2022 Jun 14;27(12):3819. doi: 10.3390/molecules27123819 (PMC9228122; doi:10.3390/molecules27123819)
Supplement: Supplementary file 1 [file molecules-27-03819-s001.zip › molecules-1686034-supplementary.pdf]

Supplementary data

Hargrave *et at*. Molecules, 2022

1. Cell cycle

Table S1: Data analysis comparing flow cytometric quantification of individual cell cycle phases across 24-hour and 48-hour timelines. * indicates a statistically significant difference between timelines. (P-value <0.05).

**MCF-7**	Timeline		Medium only	DMSO	Paclitaxel	STX3451	Radiation	Combination
**Sub-G_1**	24-hours	Mean(%)	3.22	2.94	5.64	7.82	3.11	8.45
		SD	2.02	0.90	1.52	1.35	0.83	2.11
	48-hours	Mean(%)	1.52	2.69	6.70	7.56	3.13	8.51
		SD	1.52	1.42	1.40	1.10	0.55	1.04
	P-value		0.69	0.83	0.53	0.85	0.97	0.98
**G_1**	24-hours	Mean(%)	35.50	48.01	7.23	16.74	20.31	14.3
		SD	4.53	0.13	1.43	0.06	1.21	2.40
	48-hours	Mean(%)	42.19	38.20	7.06	17.26	30.68	14.61
		SD	1.89	2.07	0.58	0.07	3.14	0.50
	P-value		0.12	0.01*	0.86	0.02*	0.01*	0.83
**S**	24-hours	Mean(%)	9.33	12.74	15.41	21.93	13.03	19.98
		SD	2.61	3.43	4.07	3.07	3.11	2.89
	48-hours	Mean(%)	4.64	4.19	6.69	14.26	5.36	13.08
		SD	1.46	1.00	0.56	0.56	0.98	1.88
	P-value		0.09	0.01*	0.03*	0.01*	0.05	0.02*
**G_2-M**	24-hours	Mean(%)	43.78	29.58	73.67	52.59	61.25	58.13
		SD	10.55	5.13	4.34	3.66	1.71	2.05
	48-hours	Mean(%)	50.73	54.11	79.49	63.20	58.46	64.69
		SD	2.08	0.21	2.43	0.62	2.12	1.31
	P-value		0.33	0.02*	0.17	0.01*	0.28	0.03*

Table S2: Data analysis of flow cytometric quantification of the cell cycle distribution in MCF-7 cells exposed to STX3451 and radiation. Cells were terminated 24-hours after radiation exposure. Mean (%) and SD of 3 biological repeats are indicated. Statistical significance (*P*-value <0.05) was calculated relative to DMSO, STX3451 and radiation (significance indicated by *).

**MCF-7**		Medium only	DMSO	Paclitaxel	STX3451	Radiation	Combination
**Sub-G_1 phase**	Mean (%)	3.22	2.94	5.64	7.82	3.11	8.45
	SD	2.02	0.9	1.52	1.35	0.72	2.12
	P-value when compared to:						
	DMSO		0.84	0.082	0.0065*	0.81	0.024*
	STX3451						0.7
	Radiation (6 Gy)						0.023*

G_1 phase	Mean (%)	35.50	48.01	7.23	16.74	20.31	14.3
	SD	4.53	0.13	1.43	0.064	1.21	2.40
	P-value when compared to:						
	DMSO		0.06	$6x10^{-4}$*	$1x10^{-5}$*	$8x10^{-5}$*	0.0025*
	STX3451						0.29*
	Radiation (6 Gy)						0.031*
S phase	Mean (%)	9.33	12.74	15.45	21.97	13.03	19.98
	SD	2.61	3.43	4.07	3.07	3.12	2.89
	P-value when compared to:						
	DMSO		0.29	0.44	0.014*	0.91	0.018*
	STX3451						0.42
	Radiation (6 Gy)						0.028*
G_2-M phase	Mean (%)	43.77	29.58	73.67	52.59	61.25	58.13
	SD	3.47	5.13	4.34	3.66	1.71	2.05
	P-value when compared to:						
	DMSO		0.044*	0.011*	0.036*	0.014*	0.018*
	STX3451						0.20
	Radiation (6 Gy)						0.24

Table S3: Statistical analysis of cell cycle distribution in MCF-7 cells exposed to STX3451 and radiation. Cells were terminated 48-hours post radiation exposure. Mean (%) and SD calculated from 3 biological repeats are displayed. Statistically significant differences (*P*-value <0.05) were calculated relative to DMSO, STX3451 and radiation (indicated by *).

**MCF-7**		Medium only	DMSO	Paclitaxel	STX3451	Radiation	Combination
Sub-G_1 phase	Mean (%)	2.54	2.69	6.69	7.55	3.13	8.51
	SD	1.86	1.74	1.71	1.54	0.67	1.47
	P-value when compared to:						
	DMSO		0.92	0.047*	0.05	0.70	0.031*
	STX3451						0.59
	Radiation (6 Gy)						0.010*
G_1 phase	Mean (%)	42.19	38.20	7.06	17.27	30.68	14.61
	SD	2.31	2.54	0.72	0.09	3.84	0.61

	P-value when compared to:						
	DMSO		0.11	$3.4x10^{-5}$*	0.0016*	0.047*	$1x10^{-4}$*
	STX3451						0.01*
	Radiation (6 Gy)						0.002*
**S phase**	Mean (%)	4.64	4.19	6.69	14.26	5.35	13.08
	SD	1.8	1.23	0.68	0.68	1.38	2.30
	P-value when compared to:						
	DMSO		0.74	0.037*	$2x10^{-4}$	0.39	$4.1x10^{-3}$*
	STX3451						0.44
	Radiation (6 Gy)						0.025*
**G_2-M phase**	Mean (%)	50.73	54.11	79.49	63.20	58.46	64.70
	SD	2.55	0.30	2.98	0.76	2.60	1.61
	P-value when compared to:						
	DMSO		0.174	0.0014*	$6x10^{-4}$*	0.11	0.0031*
	STX3451						0.22
	Radiation (6 Gy)						0.024*

Table S4: Data analysis comparing flow cytometric quantification of individual cell cycle phases across 24-hour and 48-hour timelines in MDA-MB-231 cells. Statistical significance between timelines is indicated by *. (*P*-value <0.05)

**MDA-MB-231**	Timeline		Medium Only	DMSO	Paclitaxel	STX3451	Radiation	Combination
**Sub-G_1**	24-hours	Mean(%)	0.89	1.36	1.92	4.41	2.44	4.89
		SD	0.57	1.43	0.91	3.87	2.95	4.54
	48-hours	Mean(%)	0.77	0.74	3.20	2.55	1.24	4.41
		SD	0.41	0.29	1.81	1.58	1.19	0.92
	P-value		0.78	0.50	0.41	0.50	0.55	0.90
**G_1**	24-hours	Mean(%)	56.92	60.63	16.73	50.75	28.14	26.33
		SD	3.22	2.03	2.30	0.39	5.59	2.80
	48-hours	Mean(%)	50.36	52	6.46	24.25	32.39	23.26
		SD	0.60	0.76	2.39	3.67	1.81	2.09
	P-value		0.03*	0.003*	0.009*	0.02*	0.38	0.40
**S**	24-hours	Mean(%)	8.15	9.30	9.42	16.17	15.98	16.33
		SD	2.47	2.18	3.79	4.57	8.51	4.24
	48-hours	Mean(%)	4.37	5.01	11.47	26.22	11.05	16.64
		SD	2.09	1.75	5.99	0.87	5.24	2.96
	P-value		0.10	0.049*	0.69	0.06	0.53	0.93
**G_2-M**		Mean(%)	26.36	27.87	75.05	49.44	44.96	54.80

	24-hours	SD	3.50	3.94	0.81	0.88	0.04	4.74
	48-hours	Mean(%)	44.56	42.15	72.01	62.61	66.59	54.70
		SD	2.36	2.52	2.47	5.91	2.54	1.45
	P-value		0.008*	0.008*	0.35	0.16	0.001*	0.98

Table S5: Statistical analysis of cell cycle progression in MDA-MB-231 cells exposed to STX3451 and radiation. Cells terminated 24-hours after radiation exposure. Mean (%) and SD indicated were calculated from 3 biological repeats. Statistically significant differences (*P*-value <0.05) when compared to DMSO, STX3451 and radiation are indicated by *.

**MDA-MB-231**		Medium only	DMSO	Paclitaxel	STX3451	Radiation	Combination
**Sub-G_1 phase**	Mean (%)	0.89	1.36	1.92	4.41	2.44	4.89
	SD	0.57	1.43	0.90	3.87	2.95	4.54
	P-value when compared to:						
	DMSO		0.56	0.58	0.20	0.53	0.19
	STX3451						0.89
	Radiation (6 Gy)						0.40
**G1 phase**	Mean (%)	56.58	60.62	16.73	50.75	28.14	26.33
	SD	3.22	2.03	2.30	0.39	5.59	2.80
	P-value when compared to:						
	DMSO		0.20	2×10^{-5}*	0.0075*	2×10^{-4}*	5×10^{-4}*
	STX3451						0.0066*
	Radiation (6 Gy)						0.70
**S phase**	Mean (%)	8.15	9.30	9.42	16.17	15.96	16.33
	SD	2.47	2.18	3.79	4.57	8.51	4.24
	P-value when compared to:						
	DMSO		0.51	0.96	0.043*	0.18	0.034*
	STX3451						0.97
	Radiation (6 Gy)						0.95
**G_2-M phase**	Mean (%)	26.36	27.87	75.05	49.44	44.98	54.80
	SD	3.50	3.94	7.03	0.88	0.04	4.74
	P-value when compared to:						

	DMSO	0.69	5x10^{-4}*	0.0054*	0.010*	0.006*
	STX3451					0.26
	Radiation (6 Gy)					0.10

Table S6: Statistical analysis of cell cycle distribution in MDA-MB-231 cells exposed to STX3451 and radiation. Cells terminated 48-hours after radiation exposure. Mean (%) and SD of 3 biological repeats are indicated. Statistically significant differences (*P*-value <0.05) were calculated relative to DMSO, STX3451 and radiation (indicated by *).

**MDA-MB-231**		Medium only	DMSO	Paclitaxel	STX3451	Radiation	Combination
**Sub-G$_1$ phase**	Mean (%)	0.77	0.74	3.20	2.55	1.24	4.41
	SD	0.51	0.36	2.22	1.93	1.45	1.30
	P-value when compared to:						
	DMSO		0.92	0.13	0.18	0.59	0.016*
	STX3451						0.33
	Radiation (6 Gy)						0.09
**G$_1$ phase**	Mean (%)	50.36	52	6.45	24.45	32.39	23.26
	SD	0.74	0.93	2.93	5.18	2.56	2.95
	P-value when compared to:						
	DMSO		0.076	5x10^{-5}*	0.002	0.001*	5x10^{-4}*
	STX3451						0.83
	Radiation (6 Gy)						0.08
**S phase**	Mean (%)	4.37	5.01	11.47	26.22	11.05	16.64
	SD	2.56	2.15	7.33	1.23	7.40	3.62
	P-value when compared to:						
	DMSO		0.76	0.22	0.0012	0.25	0.0087
	STX3451						0.041*
	Radiation (6 Gy)						0.32
**G$_2$-M phase**	Mean (%)	44.56	42.15	72.01	62.61	66.59	54.70
	SD	2.88	3.09	3.49	8.35	3.59	2.04

P-value when compared to:					
DMSO	0.38	0.002*	0.026*	0.0038*	0.016*
STX3451					0.32
Radiation (6 Gy)					0.055

2. Annexin

Table S7: Annexin-V analysis of MCF-7 cells 48-hours. Statistical data calculated were generated via Kaluza Analysis software version 2.1 (Miami, Florida, USA). Data were analysed using ANOVA- single factor model and a two-tailed Student's *t*-test. The averaged percentage and standard deviation (SD) of at least three biological repeats are displayed for viable cells, cells undergoing apoptosis and necrotic cells. Statistically significant differences (*P*-value <0.05) were calculated using cells exposed to DMSO. *P*-values were also calculated for the combination treatment condition when compared to individual treatments.

**MCF-7**		Medium only	DMSO	Colchicine	STX3451	Radiation	Combination
**Viable**	Mean (%)	89.73	91.94	56.37	29.22	81.43	39.68
	SD	3.47	1.23	4.34	6.27	4.46	0.86
	P-value when compared to:						
	DMSO		0.35	7.2x10^{-4}*	7.03x10^{-5}*	0.012*	1.66x10^{-5}*
	STX3451						0.12
	Radiation (6 Gy)						2x10^{-4}*
**Apoptosis**	Mean (%)	8.4	5.98	40.92	39.19	11.19	44.64
	SD	3.33	2.31	5.07	3.62	6.15	1.63
	P-value when compared to:						
	DMSO		0.34	4.07x10^{-4}*	1.79x10^{-4}*	0.24	2.70x10^{-4}*
	STX3451						0.15
	Radiation (6 Gy)						5.6x10^{-3}*
**Necrosis**	Mean (%)	1.56	2.96	11.83	23.85	6.04	10.87
	SD	3.47	1.23	2.57	6.48	2.27	5.63
	P-value when compared to:						
	DMSO		0.072	1.64x10^{-3}*	1.29x10^{-3}*	0.054	0.033*

	STX3451	0.036*
	Radiation (6 Gy)	0.16

Table S8: Annexin-V statistical analysis of MDA-MB-231 48-hours. Data calculated were analysed using Kaluza Analysis software version 2.1 (Miami, Florida, USA). Data were analysed using ANOVA-single factor model and a two-tailed Student's *t*-test. The averaged percentage and standard deviation (SD) of at least three biological repeats are displayed for viable cells, cells undergoing apoptosis and necrotic cells. Statistically significant differences were calculated using cells exposed to DMSO (*P*-value <0.05). *P*-values were also calculated for the combination treatment condition when compared to individual treatments.

**MDA-MB-231**		Medium only	DMSO	Colchicine	STX3451	Radiation	Combination
**Viable**	Mean (%)	85.23	87.10	51.22	33.84	72.43	28.96
	SD	2.4	1.13	5.90	4.90	2.32	0.26
	P-value when compared to:						
	DMSO		0.21	$1.83x10^{-4}$*	$2.01x10^{-5}$*	$2.83x10^{-5}$*	$2.85x10^{-7}$*
	STX3451						0.29
	Radiation (6 Gy)						$2x10^{-5}$*
**Apoptosis**	Mean (%)	11.93	9.91	38.23	56.53	18.38	68.47
	SD	2.12	1.31	4.24	2.27	1.07	3.82
	P-value when compared to:						
	DMSO		0.16	$1.69x10^{-4}$*	$4.73x10^{-6}$*	$5.85x10^{-5}$*	$6.95x10^{-6}$*
	STX3451						0.063
	Radiation (6 Gy)						$1.10x10^{-5}$*
**Necrosis**	Mean (%)	2.13	2.41	12.00	7.66	9.20	10.02
	SD	0.50	0.22	2.76	2.73	2.23	4.10
	P-value when compared to:						
	DMSO		0.34	$8.25x10^{-4}$*	0.011*	$9.25x10^{-4}$*	0.010*
	STX3451						0.43
	Radiation (6 Gy)						0.74

3. Clonogenic studies

Table S9: Colony formation in MCF-7 cells. Statistical data were calculated using the number of colonies as measured by manual count. Data were analysed using a two-tailed Student's *t*-test. Surviving fraction was obtained according to the plating efficiency in each treatment condition. Plating efficiency was calculated according to the number of cells seeded in each treatment condition. Surviving fraction and standard deviation are displayed for three biological repeats are displayed for the surviving fraction. Statistically significant differences (*P*-value <0.05) were calculated using cells grown in medium only as a baseline reference. *P*-values for the combination treatment compared to individual treatments were also calculated.

MCF-7	Medium only	DMSO	Etoposide	STX3451	Radiation	Combination
Surviving fraction	1	0.86	0	0.066	0.091	0.0033
SD	-	0.12	0	0.02	0.01	0.002
P-value when compared to						
Medium only	-	0.20	-	$6.35x10^{-8}$*	$4.44x10^{-9}$*	$1.21x10^{-12}$*
STX3451						0.0027*
6 Gy radiation						$5.12x10^{-5}$*

Table S10: Colony formation in MDA-MB-231 cells. A manual count was used to determine the number of colonies formed under different treatment conditions. Statistical data were calculated using these counts. Data were analyzed using a two-tailed Student's *t*-test. Surviving fraction was calculated using the plating efficiency for medium only cells and adjusting according to the number of cells seeded in each treatment condition. Average fold change and standard deviation of surviving fraction are displayed for three biological repeats. Statistically significant differences (*P*-value <0.05) were calculated by comparing treatment conditions to medium only propagated cells. *P*-values for the combination treatment cells were also compared to individual treatments.

MDA-MB-231	Medium only	DMSO	Etoposide	STX3451	Radiation	Combination
Surviving fraction	1	0.96	-	0.036	0.061	$7.1x10^{-4}$
SD	-	0.12	-	0.068	0.061	0.0012
P-value when compared to						
Medium only	-	0.55	-	$1.64x10^{-9}$*	$9.01x10^{-9}$*	$1.51x10^{-12}$*
STX3451						$9.46X10^{-4}$*
6 Gy radiation						$5.19x10^{-4}$*

4. Micronuclei quantification

Table S11: The total number of Mn in MCF-7 cells that were terminated 2- and 24-hours after radiation. Mean (%) and SD from 3 biological repeats were determined. Statistically significant differences (*P*-value <0.05) were calculated for comparisons to DMSO, STX3451 and radiation alone. Statistical significance is indicated by *.

**MCF-7**		Medium only	DMSO	Etoposide	STX3451	Radiation	Combination
**2-hour timeline**	Mean (%)	13.67	15	91	124.5	99	358.5
	SD	11.5	10.58	39.60	50.20	25.46	68.90
	P-value when compared to:						
	DMSO		0.89	0.042*	0.029*	0.012*	5x10^{-4}*
	STX3451						0.035*
	Radiation (6 Gy)						0.016*
**24-hour timeline**	Mean (%)	8	7	103	134.5	198.5	322.33
	SD	2	4.58	28.9	75.66	13.44	41.04
	P-value when compared to:						
	DMSO		0.75	4.8x10^{-3}*	0.050	1.5x10^{-4}*	1.8x10^{-4}*
	STX3451						0.033*
	Radiation (6 Gy)						0.030*

Table S12: The total number of Mn in MDA-MB-231 cells terminated 2- and 24-hours after radiation. Mean (%) and SD from were calculated from 3 biological repeats. Statistically significant differences (*P*-value <0.05) were calculated relative to DMSO, STX3451 and radiation alone (indicated by *).

**MDA-MB-231**		Medium only	DMSO	Etoposide	STX3451	Radiation	Combination
**2-hour timeline**	Mean (%)	10	13	107.67	170	92.667	384.33
	SD	2	9.165	15.177	28.28	11.37	37.17
	P-value when compared to:						
	DMSO		0.61	7x10^{-4}*	0.002*	7x10^{-4}*	7x10^{-5}*
	STX3451						6.5x10^{-3}*
	Radiation (6 Gy)						2x10^{-4}*
**24-**	Mean (%)	4.33	6.33	83	141.5	173.33	347

SD	1.15	1.52	7	23.35	24.54	27.84
P-value when compared to:						
DMSO		0.75	4.99×10^{-5}*	1.6×10^{-3}*	2×10^{-4}*	2.95×10^{-5}*
STX3451						3.4×10^{-3}*
Radiation (6 Gy)						1.3×10^{-3}*

Table S13: Number of Mn per cell in MCF-7 cells terminated 2-hours after radiation. Mean (%) and SD from 3 biological repeats are indicated for the proportion of cells containing 1-, 2-, 3-, 4-, 5-, and >5 Mn. Statistical significance is indicated by * for comparisons to DMSO, STX3451 and radiation alone (*P*-value <0.05).

	**MCF-7**		Medium only	DMSO	STX3451	Radiation	Combination
**2-hour**	0 Mn	Mean (%)	488.33	486.67	309	419.67	290
		SD	9.07	9.29	41.01	18.23	18.39
		P-value when compared to DMSO			0.0043*	0.0048*	0.0005*
		P-value when compared to STX3451					0.62
		P-value when compared to radiation					0.0044*
	1 Mn	Mean (%)	10.33	22.67	89.67	72	119.33
		SD	7.64	8.08	89.67	72	13.42
		P-value when compared to DMSO			0.017*	0.0027*	0.0003*
		P-value when compared to STX3451					0.22
		P-value when compared to radiation					0.013*
	2 Mn	Mean (%)	1	1.67	19.667	7.33	43.33
		SD	1	1.53	10.26	4.93	8.74
		P-value when compared to DMSO			0.040*	0.13	0.0012*
		P-value when compared to STX3451					0.038*
		P-value when compared to radiation					0.0034*
	3 Mn	Mean (%)	0	0	7	1	19.67
		SD	00	0	3	1	9.87
		P-value when compared to DMSO			0.016*	0.16	0.026*
		P-value when compared to STX3451					0.10
		P-value when compared to radiation					0.031*
	4 Mn	Mean (%)	0.33	0	1	0	7
		SD	0.56	0	1.73	0	4.36
		P-value when compared to DMSO			0.37	-	0.049*
		P-value when compared to STX3451					0.09
		P-value when compared to radiation					0.049*
	5 Mn	Mean (%)	0	0	0	0	2.33
		SD	0	0	0	0	1.53
		P-value when compared to DMSO			-	-	0.039*
		P-value when compared to STX3451					0.039*
		P-value when compared to radiation					0.039*
	> 5	Mean (%)	0	0	0	0	3.53

		SD	0	0	0	0	3.50
		P-value when compared to DMSO			-	-	0.16
		P-value when compared to STX3451					0.16
		P-value when compared to radiation					0.16

Table S14: Number of Mn per cell in MCF-7 cells terminated 24-hours after radiation. Mean (%) and SD are calculated from 3 biological repeats are indicated for the proportion of cells containing 1-, 2-, 3-, 4-, 5-, and >5 Mn. Statistical significance (*P*-value <0.05) is indicated by * when compared to DMSO, STX3451 and radiation alone.

	**MCF-7**		Medium only	DMSO	STX3451	Radiation	Combination
**24-hour**	0 Mn	Mean (%)	492.33	493.33	398	366.67	308.67
		SD	2.08	4.16	43.84	21.7	30.89
		P-value when compared to DMSO			0.026*	0.0006*	0.0005*
		P-value when compared to STX3451					0.07
		P-value when compared to radiation					0.056
	1 Mn	Mean (%)	7.33	6.33	101	97	127.67
		SD	2.31	3.79	43.31	11.14	22.14
		P-value when compared to DMSO			0.02*	0.0002*	0.0007*
		P-value when compared to STX3451					0.40
		P-value when compared to radiation					0.10
	2 Mn	Mean (%)	0	0	40	29	41.67
		SD	0	0	18.34	10.44	4.51
		P-value when compared to DMSO			0.030*	0.009*	$9x10^{-5}$
		P-value when compared to STX3451					0.88
		P-value when compared to radiation					0.13
	3 Mn	Mean (%)	0	0	9.5	0	4
		SD	0	0	3.54	0.58	1.53
		P-value when compared to DMSO			0.015*	0.0061*	0.0083
		P-value when compared to STX3451					0.059
		P-value when compared to radiation					0.052
	4 Mn	Mean (%)	0	0	4	2.40	3.67
		SD	0	0	2.80	0	1.53
		P-value when compared to DMSO			0.039*	0.37	0.0022*
		P-value when compared to STX3451					1
		P-value when compared to radiation					0.14
	5 Mn	Mean (%)	0	0	1.50	0	4
		SD	0	0	0.70	0	3.06
		P-value when compared to DMSO			-	-	0.13
		P-value when compared to STX3451					0.22
		P-value when compared to radiation					0.14
	>5 Mn	Mean (%)	0	0	0	0	4
		SD	0	0	0	0	3.61
		P-value when compared to DMSO			-	0.13	0.13
		P-value when compared to STX3451					0.13
		P-value when compared to radiation					0.13

Table S15: Number of Mn per cell in MDA-MB-231 cells terminated 2-hours after radiation. Mean (%) and SD were calculated from 3 biological repeats and are indicated for the proportion of cells containing 1-, 2-, 3-, 4-, 5-, and >5 Mn. * indicates statistical significance when compared to DMSO, STX3451 and radiation alone (*P*-value <0.05).

	**MDA-MB-231**		Medium only	DMSO	STX3451	Radiation	Combination
**2-hour**	0 Mn	Mean (%)	493.3	489	330	419.67	262.67
		SD	3.21	8	49.50	10.26	19.04
		P-value when compared to DMSO			0.01*	0.001*	4.5x10^{-5}*
		P-value when compared to STX3451					0.11
		P-value when compared to radiation					2.3x10^{-4}*
	1 Mn	Mean (%)	6.33	9	114	69.67	151.33
		SD	2.89	7.21	29.70	12.01	14.57
		P-value when compared to DMSO			0.0079*	0.0017*	1.1x10^{-4}*
		P-value when compared to STX3451					0.15
		P-value when compared to radiation					0.0017
	2 Mn	Mean (%)	0	2	29	8.33	58
		SD	0	2	19.31	3.06	3.06
		P-value when compared to DMSO			0.074	0.04*	0.040*
		P-value when compared to STX3451					0.11
		P-value when compared to radiation					0.0051*
	3 Mn	Mean (%)	0	0	8.67	3.5	21
		SD	0	0	7.64	2.1	6.93
		P-value when compared to DMSO			0.12	0.05	0.0063*
		P-value when compared to STX3451					0.11
		P-value when compared to radiation					0.012*
	4 Mn	Mean (%)	0	0	0	0	3.33
		SD	0	0	0	0	3.21
		P-value when compared to DMSO			-	-	0.15
		P-value when compared to STX3451					0.33
		P-value when compared to radiation					0.15
	5 Mn	Mean (%)	0	0	2	0	3.33
		SD	0	0	0	0	2.08
		P-value when compared to DMSO			-	-	0.05
		P-value when compared to STX3451					0.22
		P-value when compared to radiation					0.05
	>5 Mn	Mean (%)	0	0	0	0	4
		SD	0	0	0	0	1
		P-value when compared to DMSO			-	-	0.0022*
		P-value when compared to STX3451					0.019*
		P-value when compared to radiation					0.0023*

Table S16: The number of Mn per cell in MDA-MB-231 cells that were terminated 24-hours after radiation. Mean (%) and SD from 3 biological repeats are displayed for the proportion of cells containing 1-, 2-, 3-, 4-, 5-, and >5 Mn. Statistical significance (*P*-value <0.05) for comparisons to DMSO, STX3451 and radiation alone is indicated by *.

	**MDA-MB-231**		Medium only	DMSO	STX3451	Radiation	Combination
**24-hour**	0 M	Mean (%)	495.67	494.33	388	374.67	272
		SD	1.15	1.53	16.10	15.95	11.36

		P-value when compared to DMSO			3.39x10^{-4}*	2.09x10^{-4}*	4.69x10^{-6}*
		P-value when compared to STX3451					5.2x10^{-4}*
		P-value when compared to radiation					8.1x10^{-4}
	1 Mn	Mean (%)	4.33	5	85.67	87	151.33
		SD	1.15	1.73	19.12	14.95	14.98
		P-value when compared to DMSO			0.0019*	3.6x10^{-4}*	7.3x10^{-5}*
		P-value when compared to STX3451					0.0095*
		P-value when compared to radiation					0.0047*
	2 Mn	Mean (%)	0	0.67	21.33	30.33	50
		SD	0	0.58	3.79	2	5.86
		P-value when compared to DMSO			7.3x10^{-4}*	9.5x10-4*	3x10^{-5}*
		P-value when compared to STX3451					8.4x10^{-4}*
		P-value when compared to radiation					0.0086*
	3 Mn	Mean (%)	0	0	4.33	7	5.33
		SD	0	0	1.15	2	5.69
		P-value when compared to DMSO			0.0029*	0.0037*	0.0071*
		P-value when compared to STX3451					0.021*
		P-value when compared to radiation					0.049*
	4 Mn	Mean (%)	0	0	4.33	0.67	5.33
		SD	0	0	0.58	0.58	0.58
		P-value when compared to DMSO			0.37	8.9x10^{-5}*	0.12
		P-value when compared to STX3451					4.5x10^{-4}*
		P-value when compared to radiation					5.8x10^{-4}*
	5 Mn	Mean (%)	0	0	1.33	0	5.33
		SD	0	0	1.15	0	1.53
		P-value when compared to DMSO			0.12	-	0.014*
		P-value when compared to STX3451					0.10
		P-value when compared to radiation					0.014*
	>5 Mn	Mean (%)	0	0	0	0	3.67
		SD	0	0	0	0	1
		P-value when compared to DMSO			-	-	0.16
		P-value when compared to STX3451					0.16
		P-value when compared to radiation					0.37

5. Reactive oxygen species quantification

Table S17: Superoxide detection in MCF-7 cells treated with the various modalities. Statistical data calculated were generated via Kaluza Analysis software for Galios (Miami, Florida, USA). Data were analysed using ANOVA-single factor model and a two-tailed Student's *t*-test. The averaged fold change and standard deviation (SD) of three biological repeats are displayed for superoxide detection 24- and 48-hours after radiation. Statistically significant differences (*P*-value <0.05) were calculated using vehicle-exposed cells as a baseline. *P*-values were also calculated for the combination treatment condition when compared to individual treatments.

**MCF-7**		Medium only	DMSO	Paclitaxel	STX3451	Radiation	Combination
**24-hours**	Mean Fold change	0.90	1	1.98	1.25	1.56	1.76

	SD	0.27	0	0.31	0.14	0.30	0.24
	P-value when compared to:						
	DMSO		0.46	$2.2x10^{-3}$*	0.69	0.032*	$5.2x10^{-3}$*
	STX3451						0.039*
	Radiation (6 Gy)						0.23
48-hours post radiation	Mean Fold change	0.88	1	2.53	1.91	1.52	2.34
	SD	0.33	0	0.22	0.33	0.27	0.22
	P-value when compared to:						
	DMSO		0.78	$1.3x10^{-3}$*	0.005*	0.10	$3x10^{-4}$*
	STX3451						0.10
	Radiation (6 Gy)						0.018*

Table S18: Superoxide detection in pre-sensitized MDA-MB-231 cells. Statistical data calculated were generated via Kaluza Analysis software for Galios (Miami, Florida, USA). Data were analysed using ANOVA-single factor model and a two-tailed Student's *t*-test. The averaged fold change and standard deviation (SD) of three biological repeats are displayed for superoxide detection 24- and 48-hours after radiation. Statistically significant differences (*P*-value <0.05) were calculated using vehicle-exposed cells as a baseline. *P*-values were also calculated for the combination treatment condition when compared to individual treatments.

**MDA-MB-231**		Medium only	DMSO	Paclitaxel	STX3451	Radiation	Combination
24-hours post radiation	Mean Fold change	1.05	1	1.49	1.65	1.42	2.89
	SD	0.05	0	0.073	0.095	0.34	0.22
	P-value when compared to:						
	DMSO		0.53	$3x10^{-4}$*	$2x10^{-4}$*	0.25	$1.1x10^{-3}$*
	STX3451						0.33
	Radiation (6 Gy)						0.057
48-hours post radiation	Mean Fold change	1.01	1	3.59	2.05	1.51	2.29
	SD	0.265	0	0.30	0.66	0.32	0.13

	P-value when compared to:					
	DMSO	0.94	$1.3x10^{-3}$*	0.028*	0.044*	$2x10^{-4}$*
	STX3451 (0.75 µM)					0.83
	Radiation (6 Gy)					0.030*

6. Western blot

Table S19: Statistical analysis of ATM expression in combination treated MCF-7 and MDA-MB-231 cells 2- and 24-hours post-radiation. Mean fold change and SD are displayed for ATM expression. Data displayed were gathered from 3 biological repeats. Statistically significant differences (indicated by *) were calculated relative to DMSO, STX3451, radiation and between timelines (*P*-value <0.05).

ATM			Medium only	DMSO	Etoposide	STX3451	Radiation	Combination
MCF-7	2-hours	Mean	0.71	1.00	19.06	0.42	14.68	8.74
		SD	0.58	0.00	1.43	0.33	4.08	0.08
		P-value when compared to DMSO			$8.31X10^{-6*}$	0.015*	0.0015*	$2.68X10^{-9*}$
		P-value when compared to STX3451						$5.85X10^{-5*}$
		P-value when compared to radiation						0.18
	24-hours	Mean	0.98	1.00	9.23	0.74	2.54	2.04
		SD	0.37	0.00	1.09	0.05	0.56	0.34
		P-value when compared to DMSO			$1.98X10^{-5}$*	$3.52X10^{-4}$*	0.0015*	0.0014*
		P-value when compared to STX3451						0.014*
		P-value when compared to radiation						0.23
		P-value when compared to 2-Hrs				0.28	0.0026*	$1.19x10^{-4}$*
ATM			Medium only	DMSO	Etoposide	STX3451	Radiation	Combination
MDA-MB-231	2-hours	Mean	0.91	1	11.07	0.37	4.54	3.98
		SD	0.62	0	2.46	0.46	1.18	2.55
		P-value when compared to DMSO			$6.97X10^{-4*}$	0.034*	0.0023*	0.05
		P-value when compared to STX3451						0.19
		P-value when compared to radiation						0.80
	24-hours	Mean	0.77	1.00	5.50	0.63	1.87	0.98
		SD	0.21	0.00	1.27	0.26	0.48	0.11
		P-value when compared to DMSO			0.001*	0.033*	0.013*	0.687
		P-value when compared to STX3451						0.100
		P-value when compared to radiation						0.035*
		P-value when compared to 2-Hrs				0.45	0.034*	0.36

7. Nontumored animal toxicity assay

Nontumored Animal Toxicity Assay for S750294

Report generated on 13-Jul-2010

**EXPERIMENT:** AAZ-343 / 0 / 8B	**TUMOR:** NO CELLS	**HOST:** Athymic Nudes	**IMPLANT DATE:** 20-APR-2010
**MEMO NO:**	**SOURCE/LINE:** 0	**SOURCE:** APA	**STAGING DATE:** 20-APR-2010
**BOOK NO:**	**IMPLANT SITE:** 0	**SEX:** F	**EVALUATION DATE:** 06-MAY-2010

TREATMENT						
**Grp**	**NSC**	**Dose/Units**	**Rt.**	**Schedule**	**Death Days**	**Surv/Total Day 16**
11	D-S750294	400.00 mg/kg/dose	IP	QD X 1, Day 0	1	0/1
12	D-S750294	200.00 mg/kg/dose	IP	QD X 1, Day 0	--	1/1
13	D-S750294	100.00 mg/kg/dose	IP	QD X 1, Day 0	--	1/1

VEHICLES

**Grp** 11 ->	NSC # S750294 / 2 (Dose = 400.00)	: in 100% DMSO	(Soluble - no visible particles)	200.0 mg/ml	Inj. Vol.: 2 ul/gm body wt
**Grp** 12 ->	NSC # S750294 / 2 (Dose = 200.00)	: in 100% DMSO	(Soluble - no visible particles)	200.0 mg/ml	Inj. Vol.: 1 ul/gm body wt
**Grp** 13 ->	NSC # S750294 / 2 (Dose = 100.00)	: in 100% DMSO	(Soluble - no visible particles)	200.0 mg/ml	Inj. Vol.: 0.5 ul/gm body wt

NOTE: All treatment was administered according to exact body weight.

*Data from the National Cancer Institute (NCI, UK).
